# Lung Microbiota: From Healthy Lungs to Development of Chronic Obstructive Pulmonary Disease

**DOI:** 10.3390/ijms26041403

**Published:** 2025-02-07

**Authors:** Marija M. Stankovic

**Affiliations:** Group for Molecular Biology, Institute of Molecular Genetics and Genetic Engineering, University of Belgrade, 11042 Belgrade, Serbia; marija.stankovic@imgge.bg.ac.rs

**Keywords:** chronic obstructive pulmonary disease, lung microbiota, bacteriobiota, mycobiota, virome, host-microbiota interaction, therapy

## Abstract

Lung health is dependent on a complex picture of the lung microbiota composed of bacteriobiota, mycobiota, and virome. The studies have demonstrated that the lung microbiota has a crucial role in host protection by regulating innate and adaptive lung immunity. Chronic obstructive pulmonary disease (COPD) is an inflammatory lung disease featuring changed microbiota composition and diversity, known as a dysbiosis. The lung dysbiosis increases with the progress of COPD and during exacerbation. Two models of dysbiosis have been proposed: dysbiosis and inflammation cycles and the disturbance of bacterial interactome. Still, it is unknown if the driving factor of the pathogenesis of COPD belongs to the host or microbiota. Recently, host–microbiota and microbe–microbe interactions have been highlighted in COPD, but the mechanisms behind these interactions need further exploration. The function of the gut–lung axis is crucial for the maintenance of lung health and is affected in COPD. The application of probiotics has resulted in host–beneficial effects, and it is likely that future progress in this field will aid in the therapy of COPD. In this review, the composition of the lung microbiota, molecular mechanisms, and clinical aspects relating to host and microbiota in health and COPD are comprehensively provided.

## 1. Introduction

The respiratory tract is a part of the respiratory system enabling a vital action, that is the conduction of inhaled air to the alveoli where oxygen and carbon dioxide are exchanged with the blood. The interior of the respiratory tract is lined with respiratory epithelium serving as a defensive barrier in contact with environmental factors, such as gasses, allergens, and microbes [1]. Normally, the human respiratory tract is inhabited by a complex mixture of microbes, mainly including bacteria, fungi, and viruses which represent the microbiota of the respiratory tract [2]. By anatomical characteristics, the respiratory tract is divided into the upper respiratory tract (URT) and lower respiratory tract (LRT). The URT consists of nostrils, nasal cavities, mouth, pharynx, and a part of the larynx above the vocal cords, while the LRT consists of a part of the larynx below the vocal cords, trachea, bronchi, bronchioli, and alveoli. The anatomical structure of the respiratory tract enables the establishment of niche–specific microbe communities, dispersed like a gradient, from a high microbial load of the URT to a low microbial load of the LRT [3].

However, the human lung was primarily not considered a microbial habitat. Only by engaging specific procedures, such as culture–independent technique and whole genome metagenomic DNA sequencing method, has the existence of the lung microbiota been discerned [4,5,6]. For the study of the lung microbiota various types of samples can be employed in the analysis. For example, the collection of sputum represents a non–invasive, commonly used sampling technique, but there is a risk of sample contamination by the oral microbiota. The collection of lung biopsy and bronchoalveolar lavage fluid (BALF) are invasive approaches which are not routinely employed but are highly reliable in terms of the composition and function of the lung microbiota [2,7]. Thus, the composition of human lung microbiota can vary depending on the experimental approach. In this review, results obtained from sputum samples are predominantly presented due to their general majority.

The composition of the lung microbiota is dynamic and delineated by the immigration and elimination of microbes, as well as by their reproduction rate. Normally, microbes come into the lungs from the URT and ambient air. Studies have shown the similarity of the lung microbiota with the URT microbiota, where the oropharynx, apart from the oral cavity, seems to be the major source of the lung microbiota [8]. Moreover, *Tropheryma whipplei* is exclusively enriched in the lungs, indicating the airborne origin of the lung microbiota. However, the presence of a thin mucus layer with antimicrobial properties, low nutrition conditions, and pronounced immune activity in the lungs represent an inhospitable environment for microbial growth resulting in a relatively little reproduction. So, it has been suggested that a healthy human respiratory tract is inhabited by diverse and dynamic microbial communities [7]. Since this review is particularly focused on the microbiota of healthy and diseased lungs, microbial characteristics and effects of the other parts of the respiratory tract could be found elsewhere [5,7,8,9]. A number of studies have demonstrated that lung commensal bacteria have a crucial role in host protection by regulating innate and adaptive lung immunity [8,10]. However, the function of lung mycobiota in the maintenance of lung homeostasis has remained largely unknown. It has been suggested that the lung mycobiota is mostly interesting from clinical aspects, while the lung virome has an important role in priming and modulating host immunity.

Chronic obstructive pulmonary disease (COPD) is a common, chronic, inflammatory disease with unknown etiology. The composition of the lung microbiota in the pathogenesis of COPD has been increasingly studied, but the most of data are related to the lung bacteriobiota, while the lung mycobiota and virome are scarcely investigated [5]. However, the role of the lung microbiota in the pathogenesis of COPD is mostly unknown. The human gut and lung are functionally connected via the gut–lung axis, but its contribution to the pathogenesis of COPD is not sufficiently studied. The effects of the function of the gut–lung axis in COPD have mostly been related to the gut bacteriobiota, while the gut mycobiota and virome have been rarely investigate [11]. Elucidation of the mechanisms behind host–microbiota interactions is essential for understanding the role of the microbiota in COPD, which also could aid in the development of novel therapeutic approaches for this disease.

In this review, an overview of the current knowledge about the lung microbiota in health and COPD is provided. The role of the lung microbiota, including bacteriobiota, mycobiota, and virome, in the pathogenesis of COPD is the main focus of this review. The composition of the lung microbiota, molecular mechanisms, and clinical aspects relating to host and microbiota interactions are comprehensively provided. The role of the gut–lung axis in COPD is considered for all three gut microbiota components. The therapeutic effects of probiotics are exemplified and the development of future, personalized, and targeted therapy is proposed. Recommendations for future research are provided.

## 2. The Microbiota of the Healthy Lungs

It has been suggested that the microbiota of healthy lungs encompasses rather a transient community of microbes, than a steady microbial population. In healthy lungs, the immigration of microbes is realized by dispersion, microaspiration, and inhalation, while the elimination of microbes is governed by mucociliary clearance, coughing, and host immunity [12]. Moreover, the distribution of microbes exhibits interindividual and intraindividual specificities, which might be important for the maintenance of healthy lungs by influencing colonization resistance, microbial interactions, epithelial integrity, and lung immunity [12,13,14,15].

In healthy adults, lungs are populated by a low density of microbes (e.g., 2.2 × 10^3^ bacteria per cm^2^). However, the foundation of the lung microbiota begins very early in life, and the members of adult healthy lung bacteriobiota including *Streptococcus* spp. and *Neisseria* spp. are detected even in healthy neonatal lungs [16]. The establishment of lung bacteriobiota in early life has implicated a potential role of lung commensals in the development of the lung. It has been noticed that germ–free rodents tend to have smaller lungs [5]. Also, reduced mucus production and diminished alveoli can be normalized by *Lactobacillus* spp. lung colonization in mice [17]. This indicates that lung bacteriobiota influence lung morphology and function, but the mechanisms of their actions are not understood yet. The formation and development of lung commensals in early life have a critical impact on the maturation of the immune system by increasing the expression of genes involved in the IgA pathway in infants, which serves to establish a balance between immune recognition and tolerance [16,18]. Moreover, a study by Stein et al., conducted on Amish and Hutterite children indicated that a microbe–rich environment has a crucial role in priming and shaping the immune system and prevents the development of asthma in early life [19]. So, it has been proposed that the crosstalk between lung microbiota and the immune system has a vital role in maintaining the homeostasis of lung immunity since early life [5,20].

The interaction between host and microbiota is realized through direct contact or via microbial metabolite products. Structural and immune cells such as lung epithelial cells, macrophages, and dendritic cells, possess membrane or cytoplasmic receptors by which they perceive the presence of commensal microbes or their products, e.g., lipopolysaccharides (LPSs), glucans, chitins, etc. Microbes are capable of producing a variety of proteins, polysaccharides, lipids, peptides, amino acids, short–chain fatty acids (SCFAs), etc. [7]. Recognition of microbes by pattern recognition receptors (PRRs), such as toll–like receptors (TLRs), nucleotide–binding oligomerization domain (NOD)–like receptors (NLRs), C–type lectin receptors (CLRs), and free fatty acid receptors (FFARs), initiate signalling events that trigger immune effects [12,21]. Thus, lung microbiota disposes lung immune homeostasis and promotes immune maturation, tolerance, and resistance to various lung pathogens [19]. The effects of each species or strain are specific, implying that even a small change in the composition of microbiota can affect lung immunity.

### The Composition of the Healthy Lung Microbiota

**Figure 1:**
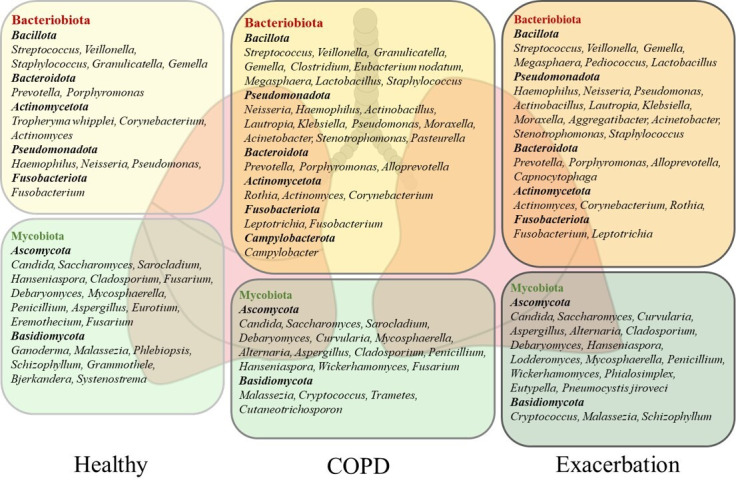
The composition of the lung microbiota in healthy subjects and patients with chronic obstructive pulmonary disease (COPD). The microbiota of healthy and COPD lungs is dominated by the bacterial phyla *Bacteroidota, Bacillota*, *Pseudomonadota*, *Fusobacteriota*, and *Actinomycetota* and the fungal phyla *Ascomycota* and *Basidiomycota*. The most commonly detected bacterial and fungal genera in sputum and bronchoalveolar lavage fluid (BALF) from healthy subjects and COPD patients are presented. The stable state of COPD is dominated by *Bacillota* and *Bacteroidota*, while *Pseudomonadota* and *Actinomycetota* increase in severe disease and during exacerbation. The fungal genera *Candida*, *Aspergillus,* and *Penicillium* feature healthy lungs, but their pathogenic effects characterize severe COPD and exacerbation. The increase in pathogens in severe COPD and exacerbation is followed by a decrease in beneficial commensals.

The most dominant bacterial phyla of the healthy human lung microbiota are *Bacteroidota* and *Bacillota*, but *Pseudomonadota*, *Fusobacteriota*, and *Actinomycetota* dominate, too [5,7,13,22,23]. The studies performed on healthy subjects by using BALF samples indicated that a core lung bacteriobiota includes the *Prevotella, Streptococcus, Veillonella, Porphyromonas, Neisseria, Fusobacterium, Haemophilus,* and *Pseudomonas* genera [13,22,23]. The composition of the healthy human lung bacteriobiota with the most commonly detected genera is shown in Figure 1. For example, a common member of the healthy lung microbiota *Prevotella* spp. is featured by weak TLR2 inflammatory properties that make colonization with these bacteria tolerable by the lung immune system [20]. A cross–sectional study conducted by Segal et al. on healthy individuals revealed that enrichment of lung microbiota with bacteria from the URT, including *Prevotella* spp., *Rothia* spp., and *Veillonella* spp., was correlated with T helper (Th) 17 inflammatory and neutrophilic profiles, and with diminished TLR4 response in macrophages. This study showed the role of commensals in activating the basal Th17 lung immune response that is protective against pathogenic bacteria and fungi [10]. Similarly, the immunomodulatory role of microbiota has been demonstrated in animal models. Aspiration of oral commensals in mice induced a MyD88–dependent Th17 immune response in the lung that protected mice against *Streptococcus pneumoniae* infection and mortality [24]. Moreover, intranasal treatment of inflammatory mouse model with exopolysaccharide (EPS) from *Bifidobacterium longum* 35624™ reduced TLR2 and interleukin (IL) 10 dependent eosinophil recruitment and expression of the Th2 markers in the lung [25]. These studies demonstrated that lung commensal bacteria have a crucial role in host protection by regulating innate and adaptive lung immunity.

The presence of fungal species in healthy respiratory tract has recently drawn increasing attention. The application of metagenomic DNA sequencing of the highly divergent internal transcribed spacer (ITS) has enabled the detection of fungal residents in healthy human lungs [26,27]. The lung mycobiota is featured by markedly poorer abundance compared with bacteriobiota and accentuated variation between healthy individuals [28,29]. The most commonly identified fungal phyla in the healthy human lungs are *Ascomycota* and *Basidiomycota*. The analysis of BALF and sputum samples has revealed the predominance of *Candida*, *Saccharomyces*, and *Malassezia* genera, but *Sarocladium*, *Grammothele*, *Penicillium*, *Cladosporium*, and *Fusarium* are also seen. A more detailed description of the healthy lung mycobiota is shown in Figure 1, [8,28,29,30]. Still, the role of lung mycobiome in the maintenance of lung homeostasis has remained largely unknown. Recent data have suggested that fungal presence, similar to bacteria, can influence immune response and inflammation, which may contribute to protection against pathogens and maintain tolerance towards beneficial commensals [8]. For example, *Candida albicans* can activate the TLR2 receptor and induce a Th2 immune response, but it can also release farnesol, a quorum–sensing mediator which inhibits cytokine production by the macrophages [31]. Also, *Aspergillus fumigatus* is capable of converting tryptophan into kynurenine resulting in immunosuppression [32]. Moreover, *Malassezia* can produce malassezin, which acts as an agonist of the aryl hydrocarbon receptor (AHR), which is a nuclear receptor with multifaceted biological functions that also influences the immune system [33]. However, very little is known about the effects of fungal commensals in a healthy lung environment, while their pathogenicity is important in clinical practice [8].

The progress in the diagnostics of viruses by deep metagenomic sequencing has empowered our knowledge about the diversity of viral species in humans. It has been shown that healthy human lungs are populated by eukaryotic viruses and bacterial virome, which is composed of bacteriophages [34,35]. A family of DNA viruses, *Anelloviridae*, encompasses the most abundant eukaryotic viruses detected in lungs, but whether anelloviruses can have a pathogenic role in human lungs remains elusive [35,36]. However, members of the *Anelloviridae* family, Torque Teno viruses (TTVs), have been connected with a decrease in T cells, and an increase in B cells and eosinophils, showing the immunomodulatory effect [34]. Also, viral families *Phycodnaviridae, Mimiviridae*, *Alloherpesviridae*, and *Poxviridae* were identified in BALF samples from healthy subjects [37]. Nevertheless, the most abundant viruses in lungs are bacteriophages, including *Siphoviridae*, *Myoviridae,* and *Podoviridae*, which infect a broad range of respiratory pathogenic bacteria such as *S. pneumoniae*, *Staphylococcus aureus*, *Stenotrophomonas maltophilia,* and *Pseudomonas aeruginosa* [36,37]. Bacteriophages have complex interactions with hosts, as their survival and fitness depend directly on their bacterial hosts. On the other side, bacteriophages can affect bacterial virulence factors, such as fitness, colonization, adhesion, invasion, and toxin production influencing the composition and burden of lung bacteriobiota, which ultimately affects host immunity [34]. In addition, lung viral latency provides a particular advantage to the host by creating a boosted basal immune status capable of controlling the subsequent infection. Thus, mice latently infected with murine herpesvirus are resistant to infection by *Listeria monocytogenes* due to the continual production of interferon (IFN) γ and activation of macrophages [38]. On the contrary, IFNγ, which is produced in response to the influenza virus in mice, suppresses bacterial phagocytosis by attenuating macrophage receptor with collagenous structure (MARCO) on alveolar macrophages and enhances susceptibility to secondary bacterial infection [39]. Similarly, the influenza virus attenuates the production of *S. aureus*–induced Th17–related antimicrobial peptides necessary for bacterial clearance in the lung [40].

Currently, the majority of published studies are related to one of the three components of the healthy lung microbiota. Although the lung bears a small number of microbes, the lung microbiota is involved in host–microbe and microbe–microbe interactions and thus is very complex. There is a need to analyze overall lung microbiota effects on healthy human lungs, by using metagenomic sequencing, multi–omics approaches and bioinformatic tools, in order to reveal the effects of the lung commensals and mechanisms implicated in the maintenance of healthy lungs.

## 3. The Pathogenesis of COPD and the Lung Dysbiosis

### 3.1. The Pathogenesis of COPD

COPD is a common respiratory disease that is characterized by functional and structural changes in the lungs usually caused by inhalation of toxic particles or gasses such as cigarette smoke. COPD is described as chronic lung inflammation, airway remodelling and emphysema causing persistent airflow obstruction and progressive lung function impairment [41]. In patients with COPD, lung inflammation is characterized by increased numbers of neutrophils, macrophages, and lymphocytes, including innate and adaptive immune actions. COPD is a heterogeneous lung disease predominantly featured by neutrophilic inflammation, but some patients demonstrate eosinophilic dominance resembling the pathology of asthma [41,42]. The course of COPD is frequently impaired by the occurrence of acute exacerbations which are followed by worsening of disease symptoms, including increased breathlessness, cough, and sputum production, as well as increased local and systemic inflammation [41,43]. Exacerbations are usually caused by viral or bacterial respiratory infection, which initiates a cascade of host inflammatory responses [42,43]. Acute exacerbations of COPD are associated with a rapid decline in lung function, higher mortality and morbidity rates, and increased healthcare expenses [43]. A detailed description of the mechanisms of the pathogenesis of COPD can be found elsewhere [42,43,44,45]. Although inflammation is thought to have the greatest contribution to the pathogenesis of COPD, the exact mechanisms of disease manifestation and progression remain still unclear.

### 3.2. The Lung Dysbiosis in COPD

Collectively, data have suggested that the composition of the lung microbiota in mild and moderate COPD patients is similar to that in healthy subjects, while the changes occur in severe disease and during exacerbations [13,46,47,48,49]. A change in the lung microbiota results from the disruption of immigration, proliferation, and elimination of microbes. In COPD a change in lung microbiota composition is known as a dysbiosis. Also, dysbiosis refers to the dominance of particular microbes in the lungs, such as pathogens overgrowth during pneumonia [50]. Dysbiosis is defined as a deviation from a normal microbial composition and is associated with unfavourable biological effects which may have clinical consequences [9]. Moreover, a considerable variation in microbiota composition has been noticed among different lung compartments in COPD [13]. Rarely, the transfer of microbes from the gut microbiota to the lung can occur via gastroesophageal reflux, lymph, and blood system which may contribute to lung inflammation and dysbiosis [7].

**Figure 2:**
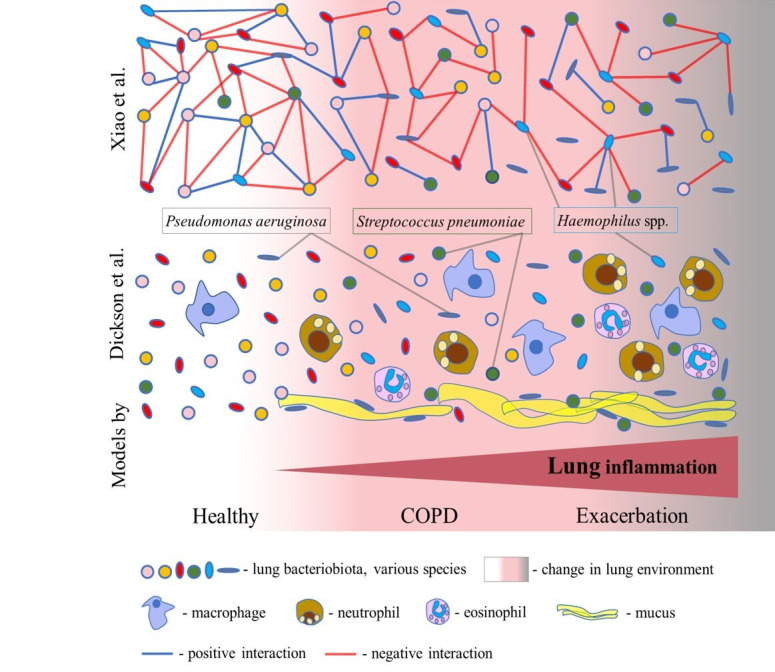
Two models of COPD dysbiosis. In the model of dysbiosis and inflammation cycles proposed by Dickson et al., lung inflammation gradually increases from COPD to exacerbation through an increase in the number of inflammatory cells. Inflammation causes a change in the lung environment and consequently a change in the composition of the bacteriobiota. Mucus favours the growth of the lung pathogens *Pseudomonas aeruginosa* and *Streptococcus pneumoniae*. A model of disturbances in the bacterial interactome from the study of Xiao et al. supposes the progressive disturbance of complex bacterial interaction networks from stable COPD to exacerbation. Bacteria realize both positive and negative interactions, leading to cooperation and competition, respectively. Herein, lung dysbiosis in COPD is characterized by reduced negative interactions in stable COPD and during exacerbation. The interactome of *Haemophilus* spp. indicates an increase in negative interactions in COPD.

Lung inflammation is a prominent feature of COPD that impacts the respiratory environment, influencing the growth of microbes and lung microbiota composition. However, the exact role of the lung microbiota in the pathogenesis of COPD is far from clear. Based on the current knowledge, lung dysbiosis in COPD can be summarized using the two models presented in Figure 2. Dickson et al. proposed a model of dysbiosis and inflammation cycles that implies a bidirectional relationship between the host and microbiota [51]. According to this model, inflammation changes the lung environment and bacterial growth, leading to dysbiosis. For example, the secretion of mucus causes a slight increase in temperature and a decrease in oxygen pressure, promoting the growth of particular microbes. Mucus converts a deprived lung environment into a nutrient–rich one, favouring the growth of opportunistic lung pathogens such as *P. aeruginosa* and *S. pneumoniae* (Figure 2). The shift towards pathogenic species and their metabolites activates more immune responses, resulting in an increase in inflammation, which brings additional changes in the lung environment (Figure 2). Dysbiosis and inflammation cycles perpetuate and progress lung inflammation and change the lung microbiota. In acute exacerbation, the lung inflammation is highly amplified, and dysbiosis is increased, which causes lung injury [14].

Recent studies have highlighted that lung dysbiosis in COPD results from the disturbance of the bacterial interactome (i.e., complex interaction networks) [15,46]. Based on these studies, the second model is proposed herein. In the comprehensive study by Xiao et al., the analysis of the interactome dynamics revealed that COPD dysbiosis is characterized by reduced antagonistic (negative) interactions, rather than a loss of diversity, in the stable state of disease and during exacerbation (Figure 2). A decrease in antagonistic interactions was associated with worse clinical symptoms, including dyspnea, decreased lung function, exaggerated neutrophilic inflammation, and a higher risk of exacerbation [15]. However, an increase in antagonistic interactions in COPD was indicated for the *Haemophilus* spp. interactome, rather than its increased abundance (Figure 2). Also, Wang et al. robustly identified *Haemophilus* spp. to have an excessively large number of negative interactions with other bacteria within the lung microbiota in COPD [46]. This suggests that *Haemophilus* spp. can act as a dominant pathogen and the main driving factor of dysbiosis in COPD. A recent study reported that the strong virus–bacterium interactions detected in the healthy gut were disrupted in patients with COPD, corroborating this model [52]. Also, an analysis of sputum samples from COPD patients identified bacterium–fungus interactions within the two clusters. In the *Aspergillus* cluster, there were connections between *Haemophilus*, *Neisseria*, *Moraxella*, *Plectosphaerella*, *Saccharomycopsis*, and *Wickerhamomyces*, while in the *Candida* cluster, connections between *Psathyrella*, *Saccharomyces*, *Phaeosphaeria*, *Peniophora*, and *Leptotricchia* were found [53]. Furthermore, a recent comprehensive surveillance performed by Lin et al. revealed enhanced inter–kingdom microbial interactions mediated by environmental exposure in the prediction and progression of COPD [54]. So, these observations demand a detailed examination of the complete lung microbiota and careful examination of the interactions between bacteria, fungi, and viruses in order to reveal critical threats to lung health and targets for future therapies.

Although significant progress has been recently made in the research on the lung microbiota, whether lung dysbiosis can cause the occurrence and progression of COPD or the microbiota simply mirrors the pathogenesis of COPD is not clear yet. Within the model suggested by Dickson et al., lung dysbiosis and inflammation follow each other and worsen with the progression of COPD and exacerbation. According to this model, we do not know whether a change in the lung microbiota can cause COPD. However, in the model that arose from the study by Xiao et al., the focus shifted to the positive and negative interactions which are related to bacterial cooperation and competition, respectively. This model offers better insight into the process of COPD dysbiosis, which is characterized by an imbalance between bacterial cooperation and competition, leading to the destabilization of the lung microbiota. Interestingly, following this model, the dominance of *Haemophilus* spp. is reflected in an increase in antagonistic interactions in COPD. However, if common pneumonia could cause COPD, the lung microbiota may be a driving factor of COPD.

## 4. The Composition of the Lung Microbiota in COPD

### 4.1. Bacteriobiota

The most abundant bacterial phyla in COPD lungs are *Bacillota*, *Pseudomonadota*, *Bacteroidota*, *Actinomycetota*, and *Fusobacteriota* characterizing the stable state and exacerbation of the disease (Figure 1) [14,55,56,57]. *Bacillota* and *Bacteroidota* are dominant during the stable state of disease, while *Pseudomonadota* and *Actinomycetota* are associated with the progress of COPD and mortality [14,46,57,58]. The diversity of lung bacteriobiota is negatively correlated with sputum level of IL8, as the prominent inflammatory mediator of lung inflammation in COPD which causes neutrophilic inflammation and mucus overproduction [46,58]. Also, a decrease in bacteriobiota diversity and richness is associated with emphysema, as a more severe presentation of disease, and immune cell infiltration [58]. Furthermore, although cigarette smoking is the main cause of COPD, and smoke is rich in highly toxic compounds, reported results have indicated that it does not seem to affect lung bacteriobiota composition [13,22].

There is a certain inconsistency among the studies reporting the composition of the lung bacteriobiota in COPD, which is most probably related to the heterogeneity of COPD, the severity of the disease, as well as the origin of samples for the analysis, such as sputum or BALF. However, regardless of the significant variation in these factors, the bacteriobiota of COPD lungs is dominated by *Streptococcus, Prevotella, Veillonella, Neisseria, Haemophilus, Rothia, Actinomyces, Leptotrichia,* and *Pseudomonas* [14,23,53,55,56,57]. A list of commonly found genera in COPD lungs, including BALF and sputum sample analyses is given in Figure 1. Above mentioned discrepancy can be exemplified with *Prevotella* spp. which is a common anaerobic commensal of healthy adult lungs with a protective role in the respiratory system [24]. The study of Xue et al. revealed the dominance of *Prevotella* spp. in connection with IL6 and Th17 lung inflammation, while the study of Hilty et al. reported a reduced abundance of *Prevotella* spp. in COPD [14,23]. Although a better selection of patients and choice of reliable sampling procedure could provide more precise results, we already have enough knowledge about the bacterial component of COPD lungs but understand mostly the effects of single relations between lung and bacteria.

The dominance of *Gemella* spp., *Granulicatella* spp., *Campylobacter* spp., and *Porphyromonas* spp. was reported in eosinophilic lung inflammation and associated with Th2 inflammatory profile [14,59]. The use of glucocorticoids in the treatment of eosinophilic COPD can lead to a change in microbiota and increase the risk of pneumonia. It has been shown that the abundance of pathogenic bacteria, such as *Haemophilus influenzae* and *S. pneumoniae* increased after treatment with inhaled corticosteroids [60]. *H. influenzae* is associated with lung and systemic neutrophilic inflammation and elevated levels of sputum IL1β and TNF in COPD patients [14,57]. Also, other studies have reported that *Haemophilus* spp. has the potential to change the composition of the lung microbiota by increasing antagonistic interactions leading to worsening of symptoms and progress of disease [15,46]. Moreover, patients with the predominance of *H. influenzae* or *S. pneumoniae* have severe disease, faster progression, increased mortality, and worse clinical prognosis [60]. Colonization of the lungs by *S. aureus* can trigger the formation of neutrophil extracellular traps (NETs) via the homocysteine–AKT1–S100A8/A9 axis, which causes lung tissue injury and lung function decline [49]. *Moraxella* spp. can also cause increased lung inflammation by affecting the Th1 pathway [14]. During the course of COPD, certain commensals with beneficial effects on lung homeostasis are depleted, such as *Prevotella* spp., while opportunistic pathogens increase in abundance, leading to a change in the bacteriobiota and progression of the disease.

There is characteristic enrichment of bacteria from the phylum *Pseudomonadota* in the lungs during COPD exacerbations [14,46,55]. Since exacerbations bear an increased risk of mortality, it is difficult to obtain BALF samples without a particular reason, and data on the lung microbiota during exacerbations are mostly based on analyses of sputum samples. Accordingly, the core lung bacteriobiota during exacerbations constitutes the *Haemophilus, Pseudomonas, Moraxella, Streptococcus,* and *Veillonella* genera. Additional genera that characterize exacerbations are given in Figure 1 [14,46,55,61,62]. Also, the expansion of pathogenic *Pseudomonadota*, followed by decreased diversity, has been associated with an increased incidence of acute exacerbations [14,46,61]. Moreover, several studies have reported that the increased abundance of *Haemophilus* spp., *Moraxella* spp., *Klebsiella* spp., *Pseudomonas* spp., and *Corynebacterium* spp. during exacerbations is strongly connected with reduced bacterial diversity and increased inflammation, correlating with the level of procalcitonin and TNF, the formation of NET complexes, and neutrophil and macrophage inflammation [14,46,62].

*P. aeruginosa* is an anaerobic pathogenic bacterium present with very low abundance in healthy lungs but found at higher concentrations in severe COPD and exacerbations. Similar to *Haemophilus* spp., *P. aeruginosa* can cause a neutrophilic host response [14]. During the progression of COPD, the proliferation of *Pseudomonas* spp. is enhanced by the inflammatory conditions of the respiratory environment, including increased levels of reactive oxygen species, reduced oxygen pressure, and the presence of the products of mucin fermentation, propionate [63]. The presence of *Pseudomonas* in the respiratory tract, similar to *Haemophilus* spp., can disrupt the composition of the lung microbiota, resulting in susceptibility to lung infections [15,46,64]. It has been demonstrated that *Pseudomonas* spp. can modulate the epigenetic mechanisms in host cells, such as ncRNA expression or host DNA methylation, in order to promote their own survival and colonization [65].

### 4.2. Mycobiota

The most abundant fungal phyla during the stable state of COPD and exacerbation encompass *Basidiomycota* and *Ascomycota*, exemplified by the dominance of the *Candida, Aspergillus, Cladosporium, Penicillium*, *Malassezia, Saccharomyces,* and *Curvularia* genera, as is presented in Figure 1 [28,30,53,55]. COPD patients are featured by personalized structures and variations in the lung mycobiota [30,55]. A multicentre longitudinal study conducted by Tiew et al. evaluated sputum mycobiome and reported a greater diversity in COPD patients than in healthy subjects [30]. In this study, there was no difference in the mycobiota composition depending on the severity of COPD, but variations at the geographic level were detected. The authors reported that COPD patients with a dominance of *Saccharomyces* spp. were associated with increased symptoms. Another study conducted by Martinsen et al. analyzed BALF mycobiomes and reported that both groups, COPD patients and controls, were dominated by *Candida*, *Malassezia,* and *Sarocladium,* but they found no differences in diversity between groups [28]. Also, the mycobiome in COPD was not affected by therapy such as the use of inhaled corticosteroids. On the contrary, a recent study reported different compositions of the mycobiota in COPD patients regarding eosinophilic inflammation. Accordingly, *Aspergillus* spp., *Gloeoporus* spp., *Irpex* spp., *Nigroporus* spp., and *Bjerkandera* spp. were associated with eosinophilic inflammation, whereas *Rhodotorula* spp., *Auricularia* spp., *Bullera* spp., and *Papiliotrema* spp. were associated with noneosinophilic inflammation in the sputum of COPD patients [66]. Both studies profiled the lung mycobiota using the standard ITS sequencing in a solid number of similarly described patients, but in the study by Martinsen et al., BALF was analyzed, while Yang et al. employed sputum samples. Since research on the lung mycobiota is still at the incipient stage, this can serve as an important prerequisite for focusing on BALF samples when analyzing the lung mycobiota. While these observations need to be clarified, they may certainly be very useful for diagnostic, prognostic, and therapeutic considerations.

The interaction between fungi and the host can occur directly or at a distance, and this interaction can have detrimental effects on the lungs, although these fungi are common members of the mycobiota in healthy lungs. For example, *C. albicans* is a well–known fungal pathogen and a major inducer of antifungal Th17 cells, which exhibit cross–reactivity with *A. fumigatus* [67]. Here, protective intestinal Th17 responses to C. *albicans* affect the airborne fungus *A. fumigatus*, which poses the risk of lung inflammatory diseases. Furthermore, the pathogenic fungus *Aspergillus* spp. that exists in healthy lungs can trigger Th1, Th2, and Th17 responses [29,68]. While the Th1 response is an effective aid in fungal clearance, the Th2 response can cause allergic lung diseases, even in healthy individuals [68]. At the respiratory mucosa level, *Aspergillus* spp. can also induce IL22, leading to the expression of antimicrobial peptides such as defensins that can influence the composition of the lung bacteriobiota, including *P. aeruginosa* [29]. However, studies on the mycobiota in COPD are still scarce, and more research is needed in order to gain better insights into the function of fungi in healthy and diseased lungs.

Up to now, the composition of lung mycobiota during acute exacerbation in COPD remains poorly explored. A decreased lung mycobiota diversity was identified during exacerbation in severe COPD patients and associated with mortality which may have prognostic value [30,61]. Moreover, during exacerbations in severe COPD patients, a loss of lung mycobiota diversity was associated with the necessity for invasive mechanical ventilation implicating a deleterious effect of mycobiota dysbiosis [61].

In a study by Tiew et al. COPD patients with the dominance of *Aspergillus* spp., *Curvularia* spp., and *Penicillium* spp. were connected with very frequent exacerbations, higher mortality, and an increase in serum IgE levels against these fungi [30]. In the same study, only *Penicillium* spp. was exclusively detected in nonsurvivor patients, but its role in COPD exacerbation remains to be fully explored. *Aspergillus* spp. sensitization in COPD was associated with increased symptoms, lung function decline, exacerbations, and bronchiectasis [30]. It has been reported that lung mycobiota during COPD exacerbations is characterized by fungal network integrity, including *Alternaria* spp., *Aspergillus* spp., *Cryptococcus* spp., *Curvularia* spp., *Lodderomyces* spp., *Malassezia* spp., *Penicillium* spp., and *Saccharomyces* spp. as the most prominent members, and that the integrity is not affected by exacerbation, antibiotics, or corticosteroids [30,61]. Also, in severe patients with exacerbations systemic corticosteroid therapy did not alter lung mycobiota or bacteriobiota composition [61]. Despite detected fungi associations with the severity of COPD and worse clinical outcomes, the real significance of fungal infection remains poorly defined in COPD.

### 4.3. Virome

Still, studies related to the analysis of COPD virome are scares with a small number of samples. However, a study by Mahomed et al., conducted on six sputum samples from South Africa reported the *Poxviridae*, *Myoviridae,* and *Siphoviridae* families as the most abundant [69]. *Siphoviridae* and *Myoviridae* represent bacteriophages and contain antibiotic resistance genes, mobile genetic elements, virulence genes and other genes that can affect bacterial life. In this study, the most prevalent virus was *BeAn 58058*, a member of the *Poxviridae* family, the relevance of which is unknown. Most of identified viruses were double–strand DNA viruses. In the study of Elbehery et al., virome analysis of BALF samples revealed *Megavirus chilensis* as the most abundant eukaryotic virus in healthy subjects and COPD patients [37]. *Moraxella, Pseudomonas,* and *Streptococcus* bacteriophages were the most dominant in COPD and during exacerbations. The abundance of virulence factors increased with the severity of the disease. One study examined virome during exacerbations of COPD using nasopharyngeal samples, which may not be relevant from the aspect of the lung virome and should be taken with precautions [70]. In this study, patients with viral pathogens had lower bacteriophages. The most common viral pathogens during exacerbations were rhinovirus, influenza, coronaviruses and parainfluenza virus. Reduced abundance of bacteriophages during exacerbations in COPD patients can affect the composition of lung bacteriobiota. Finally, no correlation was found between the virome and COPD.

Data regarding the composition of the lung microbiota are incomplete, with most of the research related to the lung bacteriobiota. Additionally, different investigations have employed different control subjects, patients with different stages of COPD, or different sampling techniques, which precludes combining the results and obtaining clear conclusions. While sputum samples are easily provided, they are prone to contamination with URT microbes. Despite this problem, sputum samples correlate with clinically relevant information such as the severity of disease, the level of inflammation, the use of antibiotics, and exacerbations, so they can also be reliably employed as a predictive tool [51]. On the other side, BALF samples are more precise but less represented, though in this case, the samples can be diluted, which can effect low–density lung microbiota analyses [7]. However, improvements could be achieved by applying standardized procedures, including appropriate controls during sampling and the exclusion of contaminations. Beyond this, the absence of uniform laboratory practices, sequencing protocols, bioinformatics, and data analysis pipelines limits the potential to carry out accurate comparative analyses or meta–analyses. Although different research questions require different set-ups and approaches, efforts should be invested in the development of standardized operating procedures that generate comparable data among various studies [5]. Moreover, recent studies have emphasized microbial interactions, which could play a prominent role in the pathogenesis of COPD, but the comprehensive work required for this type of research is missing [15,18,46,52,71,72].

## 5. The Bidirectional Gut-Lung Axis in Health and COPD

The idea of bidirectional function of the gut–lung axis is set up on the basis of indications that the gut microbiota impacts lung function, and the lung microbiota impacts gut function [11,73,74,75]. To date, the gut microbiota has been the most comprehensively studied human microbial community, with a comprehensive knowledge of its composition, structure, and function [76]. However, the role of lung microbiota is still insufficiently investigated precluding the comprehension of the function of the lung–gut axis.

The human gut is highly rich in bacteria (10^5^–10^11^ per mL), which realize important functions such as the fermentation of food, the production of vitamins, protection against pathogens, boosting of the immune system, etc. [77,78]. The most dominant bacterial phyla in the human gut include *Bacteroidota*, *Bacillota*, *Pseudomonadota*, *Actinomycetota*, and *Mycoplasmatota*, with *Bacteroides*, *Faecalibacterium*, and *Bifidobacterium* as the most prevalent genera [11,79]. The most common fungal genera in the healthy human gut are *Candida*, *Saccharomyces*, *Malassezia*, *Penicillium*, *Cladosporium,* and *Aspergillus* [80,81]. Besides bacteria and fungi, viruses are regular component of the gut microbiota in the healthy human gut, with predominance of bacteriophages, including the families *Myoviridae*, *Siphoviridae*, and *Microviridae* [52]. In comparison to the gut microbiota, the lung microbiota is featured by a marked decrease in diversity and abundance. As a richer microbial habitat, the gut microbiota powerfully affects lung immunity via interactions between structural cells, circulating cells, and microbial metabolites that trigger innate and adaptive immune responses in the lungs [74]. So, the gut epithelial cells and immune cells, such as dendritic cells and macrophages, are capable of collecting signals from the local microenvironment, including microbes and their metabolites, and priming naive B and T cells, which travel via the lymph and blood vessels to the lungs, leading to immune effects [12,82,83]. It has been demonstrated that low diversity of the intestinal microbiota in early life is correlated with asthma in childhood, suggesting a connection between the gut microbiota and the occurrence of lung disease. For example, low diversity of the gut microbiota during the first month of life was associated with asthma in childhood [84].

Similar to the generation of Th17 protective lung immunity by enrichment of the lung microbiota with oral commensals, the URT–lung and gut–lung axes create an effective lung immunity against lung infection via IL17A and granulocyte–macrophage colony–stimulating factor (GM–CSF) axis in macrophages. Actually, particular members of the URT bacteriobiota (e.g., *Staphylococcus* spp.) and intestinal bacteriobiota (e.g., *Lactobacillus* spp., *Enterococcus* spp., and *Clostridium* spp.) produce peptidoglycan that activates a NOD2–like receptor and further via IL17A–GM–CSF–ERK signalling stimulates pathogen killing and clearance by alveolar macrophages and the production of reactive oxygen species [73].

The occurrence of COPD is commonly associated with chronic gastrointestinal tract disorders, such as inflammatory bowel disease, while patients with chronic gastrointestinal tract disorders have a risk of developing lung diseases [85,86]. It has been demonstrated that patients with COPD have increased intestinal permeability [85]. A mouse model confirmed a correlation between the gut microbiota in COPD patients and increased lung inflammation, emphysema, a decline in lung function, airway remodelling, and mucus hypersecretion [87]. Moreover, a recent study reported that the gut virome in COPD subjects was different from healthy controls [52]. Despite the strong correlation between the gut microbiota, inflammation, and COPD, the relationship between various gut microbiota profiles and the severity of COPD remains unclear.

In a recent study, a *Prevotella* spp. dominant gut microbiota profile was associated with COPD and characterized by lower levels of faecal SCFAs, which were correlated with the severity of COPD [88]. Moreover, several bacteria in the gut showed abundance in association with COPD, including *Stenotrophomonas* spp., *Acinetobacter* spp., *Enterococcus* spp., *Lachnospira* spp., *Streptococcus sp000187445*, and *Haemophilus* spp. [49,89].

During exacerbation of COPD, there is a shift in the gut bacteriobiota towards an increase in the abundance of *Bacteroidota* and *Pseudomonadota* and a decrease in the abundance of the *Bacillota* and *Actinomycetota* phyla [89,90]. A study by Li et al. demonstrated a negative correlation between *Actinobacteria* spp. and the occurrence of COPD exacerbations [91]. Also, it has been proposed that *Lachnoclostridium* spp. and *Prevotella* spp. might be used as prognostic markers due to their increased abundance during COPD exacerbations [89].

Research on the gut–lung axis has mainly focused on the effects of the bacteriobiota, and there have been no studies on the gut mycobiota in COPD. Fungi represent just 0.1% of the total gut microbiota, which does not lessen their value in terms of their interactions with host immunity. Fungi elicit Th17 and regulatory T cell immunity, which control and resolve fungal infections, and Th2 lung immunity, involved in allergic responses. However, no studies on the gut mycobiota in COPD patients have been published yet [81].

It has been suggested that gut virome can have an important role in the development of COPD [52]. Truly little is known about the role of the gut virome in the pathogenesis of COPD. A rare, comprehensive study by Liu et al. conducted on Chinese revealed a decreased richness and diversity of the gut virome in patients with COPD [52]. The relative abundances of two viral families *Schitoviridae* and *Circoviridae* were significantly decreased in the COPD. Gut dysbiosis was presented with a decrease of 64 viral species including *Clostridium phage*, *Synechococcus phage*, and *Thermus phage* and an increase in *Bacteroides phage* and *Staphylococcus virus* in COPD patients. A decreased abundance of *Clostridium phage*, *Myoviridae sp*., *Synechococcus phage*, and *Paracoccus phage* was associated with decreased lung function in patients. Additionally, bacteriophage–bacterium and virus–bacterium interactions were decreased in COPD. These results implicate a diagnostic potential of the gut virome in COPD [52].

## 6. The Therapy of COPD

COPD is the fourth leading cause of death worldwide, causing approximately 5% of all global deaths. Patients with COPD experience an impaired quality of life, which is usually associated with an accelerated decline in lung function and progressive impairment of physical performance. Acute exacerbations are associated with significant mortality and health and socioeconomic burdens. Despite this, in recent decades, there have not been significant improvements in the therapy for COPD, which is mostly inefficient in controlling lung inflammation or infection, particularly in severe cases of the disease. The mechanisms of pathogenesis remain poorly understood, limiting the development of effective therapies. Respiratory infections are a major cause of the burden in COPD. However, according to the Global Initiative for Chronic Obstructive Lung Disease (GOLD) standard, COPD is defined as a preventable and treatable disease. So, there is an urgent need for the application of novel approaches with alleviating effects on the pathogenesis of COPD.

Numerous studies, as reviewed in this paper, have indicated that changes in the lung microbiota play an important role in the pathogenesis of COPD. So, the development of targeted anti–inflammatory and anti–infective therapies corresponding to particular microbiota profiles with alleviating effects on the symptoms of the disease may be a promising approach in the treatment of COPD. However, elucidation of the mechanisms behind host–microbiota interactions would be critical for the development of efficient therapeutic approaches in COPD. Also, lung microbiota profiles can be employed in the development of diagnostic tools that could aid in the prevention or prediction of disease, which could be critical in severe COPD and exacerbations.

**Figure 3:**
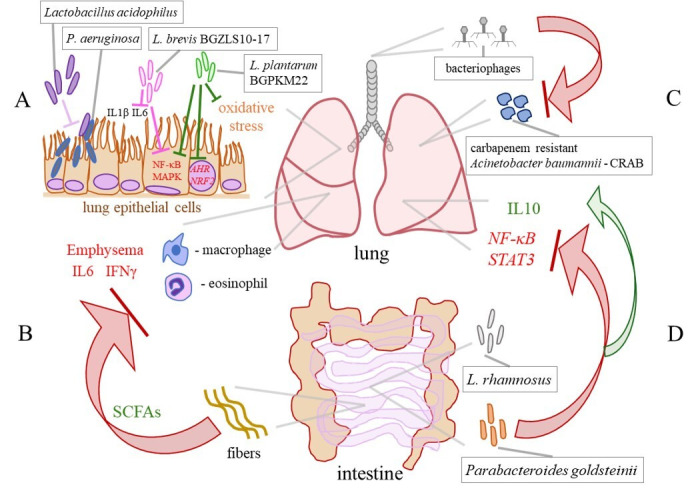
Selected effects of probiotics in COPD. (**A**) *Lactobacillus acidophilus* prevents the adhesion of *P. aeruginosa* to lung epithelial cells. *L. brevis* BGZLS10-17 and *L. plantarum* BGPKM22 attenuate the LPS–induced expression of the *interleukin (IL) 8* and *monocyte chemoattractant protein (MCP) 1* genes and the activation of nuclear factor–κB (NF–κB) and mitogen–activated protein kinases (MAPKs) in the bronchial epithelial cells. Heat–killed BGPKM22 possesses antioxidant activity and inhibits the cigarette smoke–induced expression of the *aryl hydrocarbon receptor* (*AHR)* and *nuclear factor erythroid 2 related factor 2* (*NRF2*) genes. (**B**) A high–fibre diet, via stimulation of the production of short–chain fatty acids (SCFAs), leeds to a decrease in macrophages, lymphocytes, IL6, and IFNγ in the BALF and preventes the progression of emphysema in a mouse model. (**C**) Bacteriophage therapy efficiently eliminates a carbapenem–resistant *Acinetobacter baumannii* (CRAB) from the lung of a COPD patient. (**D**) Via the lung–gut axis, *L. rhamnosus* reduces the expression of *NF–κB* and *signal transducer and activator of transcription* (*STAT*) *3* and increases the levels of IL10 in the lungs. An intestinal commensal *Parabacteroides goldsteinii* inhibits NF–κB activation in the lungs by acting through the gut–lung axis.

The field of probiotic applications is expanding rapidly, which could be highly beneficial for the management of COPD and the development of novel targeted therapies. In an in vitro study conducted by Batoni et al., *Lactobacillus acidophilus* was efficient in preventing the adhesion of *P. aeruginosa* to lung epithelial cells and significantly reduced the release of proinflammatory cytokines IL1β and IL6 from human mononuclear cells stimulated with *P. aeruginosa*. These observations point to *L. acidophilus* as a candidate for the development of treatment for better control of *P. aeruginosa* lung infections, as depicted in Figure 3A [92]. In our study, *L. brevis* BGZLS10–17 and *L. plantarum* BGPKM22 effectively reduced the expression of proinflammatory mediators in LPS–stimulated bronchial epithelial cells. The strains attenuated LPS–induced nuclear factor–κB (NF–κB) nuclear translocation and reduced the activation of the p38, ERK, and c–jun amino–terminal kinase (JNK) signalling cascade (Figure 3A). So, BGZLS10–17 and BGPKM22 could be promising candidates for balancing the lung immune system in COPD patients and replenishing diminished lung commensals, preferentially through intranasal application [93]. Furthermore, we showed that heat–killed (HK) BGPKM22 possesses antioxidant activity against cigarette smoke, resistance to hydrogen peroxide, and free radical–neutralizing activity. HK BGPKM22 successfully inhibited the cigarette smoke–induced expression of the *AHR* and *nuclear factor erythroid 2 related factor 2* (*NRF2*) genes, which could be exploited in the treatment of lung pathologies featuring elevated oxidative stress and proliferation (Figure 3A) [94].

An in vivo study emphasized the significance of the gut–lung axis in the attenuation of the occurrence of emphysema via modulation of the gut microbiota. In work by Jang et al., the modulation of the gut microbiota through faecal microbiota transplantation and a high–fibre diet attenuated lung and systemic inflammation, by decreasing macrophages, lymphocytes, IL6, and IFNγ in the BALF and prevented alveolar destruction and cellular apoptosis in a mouse model of emphysema (Figure 3B). A microbiota analysis revealed that in treated mice, the families *Bacteroidaceae* and *Lachnospiraceae*, involved in fibre metabolism and the production of SCFAs, were abundant. This finding provides a relatively simple approach to preventing and delaying COPD progression, as well as an insight into a novel therapeutic strategy for subjects with emphysema [95]. In addition, the SCFAs propionate and butyrate were capable of restoring the integrity of the bronchial epithelial cells, highlighting their potential clinical applications in diseases such as COPD [96]. A small pilot study in COPD patients demonstrated the efficacy of nutritional interventions that target the gut microbiota, showing that the supplementation of COPD patients with inulin could improve disease outcomes [97]. Another study by Carvalho et al. showed that oral feeding with *L. rhamnosus* attenuated inflammation in the airways and lung parenchyma, reduced inflammatory cell infiltration and lung tissue remodelling, and induced an anti–inflammatory effect in a cigarette smoke–induced COPD model in mice. In this study, by acting through the lung–gut axis, *L. rhamnosus* reduced the production of the proinflammatory mediators *NF–κB*, *signal transducer and activator of transcription (STAT) 3*, and *metalloproteases,* and increased the levels of IL10 and *suppressor of cytokine signalling* (*SOCS*) *3* (Figure 3D) [98]. Moreover, the intestinal commensal *Parabacteroides goldsteinii* has the capability to block NF–κB activation and inhibit lung inflammation by acting through the gut–lung axis (Figure 3D) [99].

Probiotics are commonly applied orally, but in view of lung diseases, an enhanced effect of intranasal application would be expected. An important component of bacterial cell walls is the EPS layer which, when purified from *B. longum* 35624™, replicates beneficial effects. Intranasal exposure of an inflammatory mouse model to EPS led to a decrease in the recruitment of eosinophils to the lungs and the expression of Th2 markers that was dependent on TLR2 and IL10. This study showed that EPS could be used as a novel therapeutic molecule to reduce the eosinophilic inflammation in COPD patients [25]. Interestingly, our recent research showed that the strain BGPKM22 developed a thicker EPS layer in interaction with human bronchial epithelial cells from a healthy donor than from a COPD patient [100]. This indicates that health status can affect EPS synthesis and deprive the host of probiotic effects. In this case, purified EPS can secure the host benefits of a curative matter regardless of health status. In addition, it has been demonstrated that intranasal treatment in an emphysema mouse model with *L. salivary* and *L. oris* changed the lung microbiota and increased indole–3–acetic acid in the BALF, preventing a decline in lung function and the development of emphysema in mice [101].

Overuse of antibiotics in clinical medicine has led to the spread of multidrug–resistant lung pathogens, which is a global problem. So, bacteriophage therapy has been extensively studied as an efficient and safe alternative to antibiotics for lung infections. For example, due to an increase in multidrug–resistant lung pathogen *P. aeruginosa*, there is a need for alternative treatments. Accordingly, it has been demonstrated that bacteriophage therapy is an effective treatment against chronic *P. aeruginosa* lung infections, that is even efficient against biofilm formation [102]. As it has already been mentioned lung colonization with *S. aureus* leads to lung function decline. It has been shown that intranasal application of *S. aureus* bacteriophage alleviated lung function decline in emphysema mice [49]. Bacteriophage therapy is targeted therapy against a particular microbe that prevents significant perturbations of the remaining members of the microbiota which could be employed for precise editing of the lung microbiota in COPD [49]. Furthermore, Tan et al. reported successful personalized bacteriophage therapy for a carbapenem–resistant *Acinetobacter baumannii* (CRAB) lung infection in a patient with COPD (Figure 3C). The therapy was well tolerated and resulted in the clearance of the lung pathogen, and clinical improvement of lung function in the patient [103]. So, a safe and effective way to recover balance in disturbed lung microbiota in subjects with COPD could be the application of personalized bacteriophage–targeted therapy.

Important insight has been gained regarding the nature, function, and significance of the lung microbiota in the occurrence, progress and response to therapy in COPD. The growing evidence about the significance of host–microbiota and microbe–microbe interactions and their functional assessments in the pathogenesis of COPD requests additional data processing. Integration of next–generation sequencing (NGS) and multi–omics datasets would allow a holistic assessment of COPD endophenotypes and complex microbial profiles in this disease. Identifying specific microbes, their profiles, and the metabolic signatures associated with COPD opens up potential for the development of new therapies and prognostic applications [104]. So, the use of NGS and multi–omics integration approaches in identifying patients with high risk and a poorer prognosis may aid in stratifying the therapy, but there is no such a practice yet. For example, COPD dysbiosis dominated by a *Haemophilus* profile is associated with a poorer prognosis, while dominance of *Prevotella* relates to better clinical outcomes. Despite the promise and potential of these approaches, the large amount of data generated by such technologies can be challenging to analyze and interpret, and currently, there is a lack of standardized methods to address this [104]. Finally, the idea of manipulating the microbiota and restoring a healthy ecosystem has happened to be successful in the therapy for gastrointestinal diseases, while this holds potential for COPD and is hopeful for its future application.

## 7. Conclusions

Research on the lung microbiota provides a great opportunity in the search for mechanisms implicated in the pathogenesis of COPD which may enable the development of adequate therapy for this still incurable disease. Although there is a significant amount of data on the lung bacteriobiota, our knowledge about the lung mycobiota and virome is mainly incomplete, which leaves a gap in our understanding of the complex microbial ecosystem of the lungs. More systematic studies that address the complete structure of the microbiota and its function in healthy and COPD lungs would provide better insight into the role of the lung microbiota in the maintenance of health and pathogenesis of COPD.

Recent studies have pointed out the crucial role of interactions between the host and microbiota in the occurrence and progression of COPD, which deserves further investigation. The dynamic nature of the lung microbiota demands the use of multi–omics approaches to delineate the complex microbial communities that exist in the lungs of COPD patients better. Combining several multi–omics in integration with clinical features may offer better predictions of patients’ outcomes, which may be critical in severe COPD and during exacerbations.

The gut–lung axis holds great potential in terms of the application of probiotics, the efficiency of which has already been indicated. While certain successes have been achieved regarding the use of probiotics and personalized targeted therapy in COPD, the use of novel technologies and clinical studies is expected to advance the development and application of safe, effective, and personalized treatments based on microbes in the future.

To gain better insights into the mechanisms of COPD pathogenesis, genomic, proteomic, lipidomic, and metabolomic assessments should be employed. However, these omics data are characterized by high integral dimensionality and data heterogeneity, so they still demand extensive analysis and elucidation, which will require the development of adequate approaches to the integration and analysis of said data.

## 8. Future Directions

Although significant moves in deciphering the human lung microbiota have been achieved, the majority of data are still related to the composition of bacteriobiota, while mycobiota and virome are largely neglected. Apart from that, studies have indicated controversies in the obtained results which need to be properly addressed. One of the reasons for that can be prescribed to marked heterogeneity of COPD patients, so studies with a better selection of patients would ultimately solve this issue. Large, multicentre studies with well–defined patients and healthy subjects, application of standardized sampling procedure, and analysis focused on complete microbiota would surely bring important information about the role of the lung microbiota in COPD in science and medicine.

Recent studies have highlighted the importance of microbial interactions in the occurrence and progress of COPD which brought a new hope for finding a real value of the lung microbiota in this disease. Since host–microbiota and microbe–microbe interactions affect the pathogenicity of bacteria, fungi, and viruses, which may have deleterious synergistic effects, integrated biological approaches are necessary for the scrutiny of molecular mechanisms behind these interactions. Understanding the mechanisms behind host–microbe and multi–kingdom interactions could be advanced by combining NGS and multi–omics techniques. Moreover, the identification and detailed analysis of particular members of the lung microbiota and microbial profiles with profound effects on the host and microbial community would lift research on this topic to another level.

Some of the research results, presented here, are promising regarding the diagnostic procedures, which particularly relate to severe patients with COPD. The function of the gut–lung axis has already indicated significant possibilities for the improvement of symptoms in COPD by using probiotics and nutritional approaches, and it would be expected to progress more by using next–generation probiotics. Also, microbiota restorative therapies have been suggested in COPD, but their development and application are still difficult due to the lack of understanding the lung dysbiosis, as well as the mechanisms of COPD pathogenesis. Nonetheless, the success of such an approach would be possible as much as the driving factor of COPD pathogenesis lies in the lung microbiota, but not in the host.

## Data Availability

All data generated or analyzed during this study are available from the corresponding author by request.

## Abbreviations

AHR: aryl hydrocarbon receptor; BALF: bronchoalveolar lavage fluid; CLRs: C–type lectin receptors; COPD: chronic obstructive pulmonary disease; CRAB: carbapenem–resistant *Acinetobacter baumannii*; EPS: exopolysaccharide; ERK: extracellular signal–related kinase; FFARs: free fatty acid receptors; GM–CSF: granulocyte–macrophage colony–stimulating factor; GOLD: Global Initiative for Chronic Obstructive Lung Disease; IFN: interferon; IL: interleukin; ITS: internal transcribed spacer; JNK: c–jun amino–terminal kinase; LPSs: lipopolysaccharides; LRT: lower respiratory tract; MAPKs: mitogen–activated protein kinases; MARCO: macrophage receptor with collagenous structure; MCP: monocyte chemoattractant protein; NETs: neutrophil extracellular traps; NF–κB: nuclear factor–κB; NGS: next–generation sequencing; NLRs: nucleotide–binding oligomerization domain (NOD)–like receptors; NRF2: nuclear factor erythroid 2 related factor 2; PRRs: pattern recognition receptors; SCFAs: short–chain fatty acids; SOCS: suppressor of cytokine signalling; STAT: signal transducer and activator of transcription; Th: T helper; TLRs: toll–like receptors; TTVs: Torque Teno viruses; URT: upper respiratory tract
